# Mechanism of Detecting the Construction Quality of a Diaphragm Wall by an Infrared Thermal Field and Engineering Application

**DOI:** 10.3390/ma16031052

**Published:** 2023-01-25

**Authors:** Jianxiu Wang, Pengfei Liu, Jian Hu, Weiqiang Pan, Yanxia Long, Ansheng Cao, Huboqiang Li, Yuanwei Sun

**Affiliations:** 1 College of Civil Engineering, Tongji University, Shanghai 200092, China; 2 Key Laboratory of Geotechnical and Underground Engineering of Ministry of Education, Tongji University, Shanghai 200092, China; 3 Shanghai Tunnel Engineering Company Co., Ltd., Shanghai 200082, China

**Keywords:** foundation pit, diaphragm wall, leakage, temperature field, infrared thermography

## Abstract

During underground space exploitation in the urbanization process, numerous foundation pits were constructed where a diaphragm wall was often used as a retaining structure and waterproof curtain. Due to complicated engineering geological conditions or improper construction, diaphragm walls and wall joints often exhibit quality defects. Groundwater leaked from these quality defects to foundation pits during excavation, endangering the safety of the pit and surrounding facilities. The current leakage identification of the underground retaining structure was performed by artificial visual detection, which cannot satisfy the engineering requirement. The temperature field in the leakage area of the diaphragm wall was different from other areas. The leakage wall imaging system using a thermal imager was efficient in visualizing leaking, which was not visible to the naked eye. In this study, infrared thermal imaging technology was introduced in potential leakage detection for the diaphragm wall of a foundation pit. The infrared radiation characteristics of the diaphragm wall leakage and the potential leakage parts were studied through laboratory simulation tests and on-site detection methods. The maximum temperature appeared at the water outlet and the surface of the defect with hidden defect, and the temperature field was symmetrically distributed along the cross-section direction. In the potential leakage area, the temperature difference at the penetration point was 23.4 °C when the initial water pressure was 10 kPa. The temperature difference at the penetration point was 21.8 °C when the initial water pressure was 30 kPa. In the field test, the maximum temperature difference between the leakage area and the surrounding wall was 4.5 °C. The study can provide a reference for similar engineering.

## 1. Introduction

In recent years, with the continuous development of China’s economy, people’s demand for underground space has been growing in the direction of increasing area, depth, and scale [1]. At the same time, due to the improper construction of urban underground space, underground diaphragm walls and wall joints exhibit quality defects, and water leaks from these quality defects to the foundation pit during its excavation [2,3]. As a result, the foundation pit retaining structure and the underground pipeline are damaged, thereby endangering the safety of public facilities [4,5].

In addition to strengthening the research on the construction process and methods of diaphragm walls, enhancing the monitoring of the defects and leakages of the diaphragm walls is essential to determining the positions of the defects and leakages in time and accurately [6,7]. As a result, the occurrence of dangerous situations can be effectively controlled, and corresponding treatment measures can be implemented in a timely manner in accordance with the location and extent of leakages. Therefore, accurate and timely determination of defect and leakage positions is crucial to underground engineering safety construction [8,9].

However, the leakage identification of the underground retaining structure was still performed based on artificial visual detection. The efficiency of manual detection was low during excavation. Sometimes it was difficult to find the location of the leakage source without an obvious leakage source. For complex leakage in diaphragm walls, destructive methods were often used to detect the source of seepage, which required a large amount of work. It was time-consuming and expensive, resulting in great waste. The leakage area with high water content exhibited a different temperature-varying pattern from the normal area due to the obvious difference in specific heat capacity between water and diaphragm walls. Scanning and identifying temperature field anomalies through infrared thermal imaging provided a new idea for identifying leakage in diaphragm walls. The infrared thermography non-destructive testing (ITNDT) method is a non-contact detection method widely used in water leakage identification at present. Compared with traditional manual detection methods and other methods, the measurement results of ITNDT were more intuitive, more objective, and more accurate [10,11]. Clark et al. [12] utilized ITNDT to detect the concrete leakage of several concrete bridges in the UK and applied ITNDT to detection in areas with low external temperatures in Europe; they achieved satisfactory results. Takahide et al. [13] obtained the variation rule of the internal mass defects of concrete with the buried depth and geo-metric size of defects, and a quantitative experimental study was conducted on the internal damage detection of concrete under two thermal excitation processes by using ITNDT. Toshihiro et al. [14] employed ITNDT to conduct a non-destructive detection of water leakage in a tunnel lining and provided a basis for infrared thermal field detection of water leakage in concrete. Zhou et al. [15] detected the water seepage of a building structure with an infrared thermal imager and proved the practicability and scientificity of infrared thermal image detection technology in the process of building leakage inspection. To realize the efficient detection and automatic identification of embankment leakage, Zhou et al. [16,17] proposed a detection technology for infrared thermal images carried by drones; the technology has good applicability and generalization ability for leakage detection and identification. Zhou et al. [18] conducted a model test and reported that the infrared radiation temperature at the leakage point is lower than the temperature of the background area; they also proposed an earth dam leakage discrimination index. Zumr et al. [19] combined drones with thermal imaging technology and applied geophysical methods to detect and locate the temperature distributions of small earth dam boundaries, potential erosion, cavities, and places with high water contents. Peter et al. [20] studied the leakage of 27 pipelines in Melbourne by using infrared thermography and developed a passive leakage detection method for buried network pipelines with small diameters by using infrared thermography. The abovementioned studies have shown that thermal imaging technology has been used to determine the quality of bridge concrete and dam bodies, examine pipeline leakages, and for building detection. However, the existing literature indicates that ITNDT has few engineering applications in the field of water leakage detection in foundation pit engineering [21,22]. In addition, the ITNDT leakage technology mainly focused on the occurrence of leakage instead of the potential leakage area.

In this study, the influence of temperature field distribution on a concrete surface under different seepage water temperatures and pressures was studied through laboratory experiments. The infrared radiation temperature characteristics of a deep foundation pit project at the Shanghai World Expo and the underground diaphragm wall seepage and leakage hidden dangers of Hanzhong Road Station’s foundation pit project on Line 13 in Shanghai were studied using the method of static shooting. Infrared radiation temperature images of the diaphragm wall joint were collected and analyzed after sealing the water leakage. The ITNDT leakage detection method adopted in this paper can accurately identify the water seepage outlet point of the diaphragm wall and predict the potential leakage area which was not visible to the naked eye. The results can provide theoretical guidance for nondestructive leakage detection of underground engineering.

## 2. Materials and Methods

### 2.1. Test Equipment

**Figure 1:**
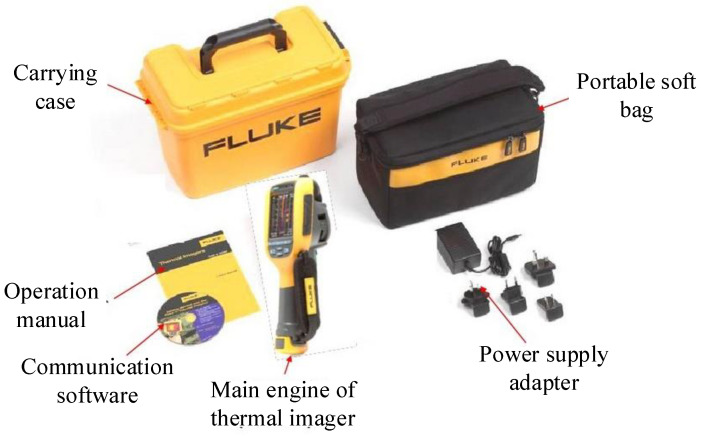
Infrared thermal imager.

To guide the infrared thermal imager in detecting concrete leakage in underground engineering, this study conducted a laboratory simulation test on in frared detection of water leakage in concrete. The rules of infrared thermal images corresponding to different leakage states and environments were explored, as shown in Figure 1. The test used the Ti110 thermal imager launched by FLUKE Company (Fluke Corporation, Everett, WA, USA). The imager has several functions, such as laser aiming, lighting, electronic compass, emissivity correction, transmittance correction, and background temperature compensation. The resolution is 160 × 120, and automatic and manual focus are combined for convenient and fast infrared detection.

### 2.2. Basic Principle of Infrared Thermal Imaging Nondestructive Testing for Underground Engineering

**Figure 2:**
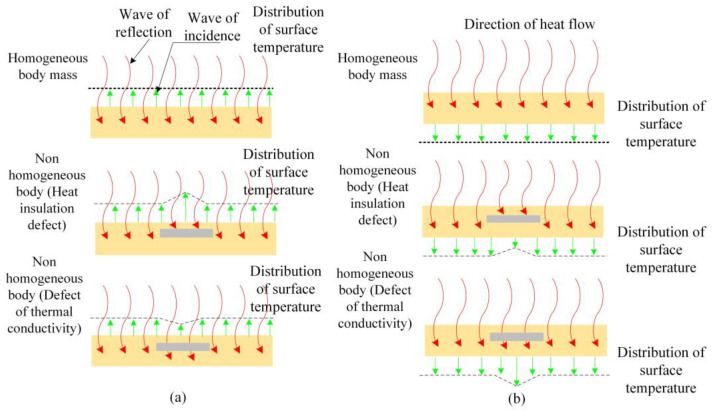
Schematic diagram of the working principle of infrared detection of water leakage and potential leakage area; (**a**) The heat source and detector are on the same side of the medium; (**b**) The heat source and detector are on both sides of the medium.

Infrared rays are electromagnetic waves that are shorter than microwave wavelengths and longer than visible light wavelengths in the electromagnetic spectrum, and their wavelength range is 0.75–1000 um. Infrared rays and electromagnetic waves have common characteristics; they propagate in space in the form of transverse waves and travel at the same speed as light does in a vacuum [23,24]. Infrared radiation is one of the most common forms of electromagnetic radiation in nature. Any object with a temperature higher than absolute zero (−273.15 °C) is a source of infrared radiation, which radiates infrared rays continuously [25,26,27]. A schematic of the working principle of infrared detection technology for defect and leakage detection is shown in Figure 2.

### 2.3. Test Scheme

This work studied the temperature field distribution of concrete samples when water seepage and its hidden dangers occurred. The tests were divided into two groups when studying the water leakage and the hidden danger of water leakage in concrete samples. The first group examined the influence of water pressure change on the surface temperature field distribution of a concrete test block. At this time, the temperature of the leaking water was unchanged at 45 °C, and the hydraulic pressures of the leaking water were 10, 20, and 30 kPa. The second group examined the influence of the change in water temperature on the surface temperature distribution of a concrete sample. At this time, the hydraulic pressure of the seepage water was unchanged at 30 kPa, and the temperatures of the seepage water were 35 °C, 40 °C, and 45 °C. The test condition is shown in Table 1.

### 2.4. Experiment Procedure

**Figure 3:**
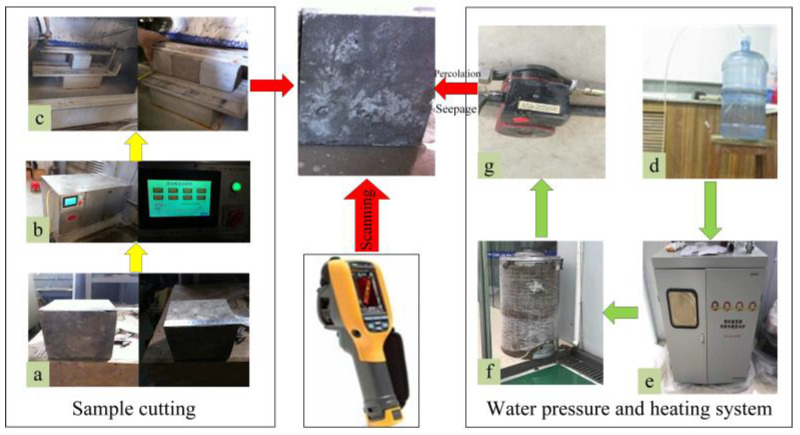
Flow chart of temperature monitoring test in permeation field; (**a**) C25 concrete sample; (**b**) Rock cutter; (**c**) Cut test block; (**d**) Seepage water; (**e**) Electromagnetic heating furnace; (**f**) Heat preservation water tank; (**g**) Booster water pump.

In this test, the situation of a 10 mm hidden defect was simulated under different water temperatures and water pressures. Through a self-made water leakage simulation system, the hidden defects were heated behind the concrete specimen, and the data detected by the infrared thermal imaging camera reflected the temperature field distribution on the surface of the concrete specimen. The test system was mainly composed of sample cutting, infiltration water heating and pressurizing systems, and an infrared thermal imaging system (Figure 3). The test was performed in a closed laboratory environment with a constant temperature and humidity, and the test steps were as follows:

(1) The self-made water leakage simulation system was assembled, and the cut concrete specimen was fixed on the bracket. The concrete sample used in the test was C25, and the finished test block had a size of 150 mm × 150 mm × 150 mm (Figure 3a). The prepared concrete sample with the strength of C25 was cut by an XGDQ-1/4 P rock cutter (Figure 3b, Jinan Puye Electromechanical Technology Co., Ltd., Jinan, China). The preparation process of the specimen is shown in Figure 3c. The level of the bracket was adjusted so that the concrete specimen was angled at 90° relative to the horizontal plane.
(2) The water was heated to the required temperature under each experiment condition (i.e., 35 °C, 40 °C, and 45 °C); the heating system was composed of seepage water (Figure 3d), frequency conversion electromagnetic heating furnace (Figure 3e), heat preservation water tank (Figure 3f), and 320 W supercharged water pump (Figure 3g). The distribution of the concrete surface temperature field corresponding to different hidden defect depths was simulated.
(3) The water pressure of water leakage was controlled by the self-made water-leakage control system. The water pressure was adjusted to 10, 20, and 30 kPa in accordance with the requirements of each experiment condition.
(4) The infrared thermal imaging camera was initialized, the equipment was adjusted to be perpendicular to the surface of the concrete specimen, the simulation system was operationalized, and infrared temperature field data on the surface of the concrete specimen were collected.

## 3. Results and Discussions

### 3.1. Analysis of the Water Seepage Simulation Test on Concrete Samples

(1) Influence of seepage water pressure on the surface temperature field

**Figure 4:**
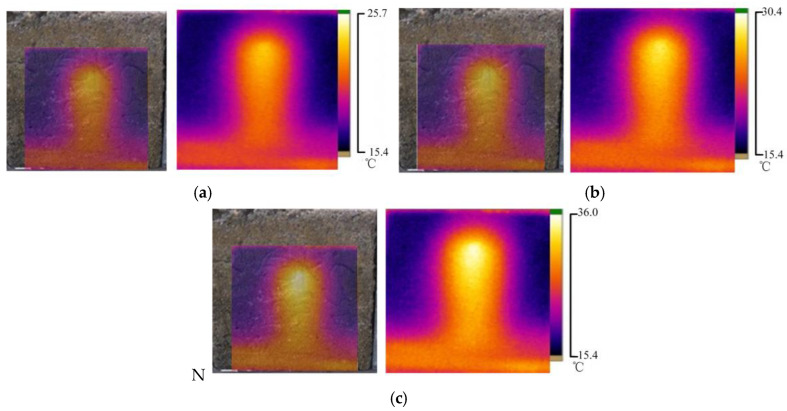
Temperature field distribution of concrete surface under different leakage water pressures; (**a**) The water pressure is 10 kPa; (**b**) The water pressure is 20 kPa; (**c**) The water pressure is 30 kPa.

To study the influence of the change in leakage water pressure on the temperature field distribution on the surface of the concrete specimen, three experimental conditions were set to simulate the corresponding variation law of water pressure and concrete surface temperature field. The water temperature in the three working conditions was 45 °C, and the seepage water pressures were 10, 20, and 30 kPa. The temperature of the concrete specimen was similar to the environment temperature of 16 °C. The temperature field distribution on the concrete surface under different leakage water pressures is shown in Figure 4.

Figure 4 indicates that the distribution law of the infrared radiation temperature field on the surface of the concrete sample was similar under the same water temperature and different water pressures. The high-temperature area was distributed in a columnar shape, and at the bottom of the concrete, the temperature was relatively high due to the flow of seepage water. With the increase in seepage water pressure, the surface heat radiation temperature of the concrete sample also increased, which was due to the fact that high-temperature water seeps rapidly under high-pressure conditions, resulting in a relatively high surface temperature.

**Figure 5:**
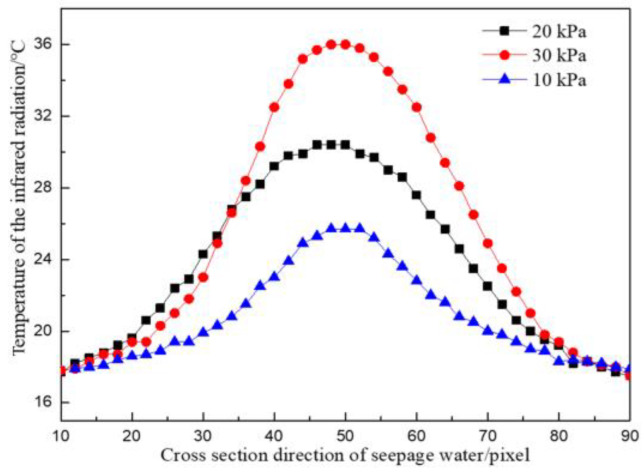
Temperature distribution curves along the cross-section direction of water flow under different working conditions.

**Figure 6:**
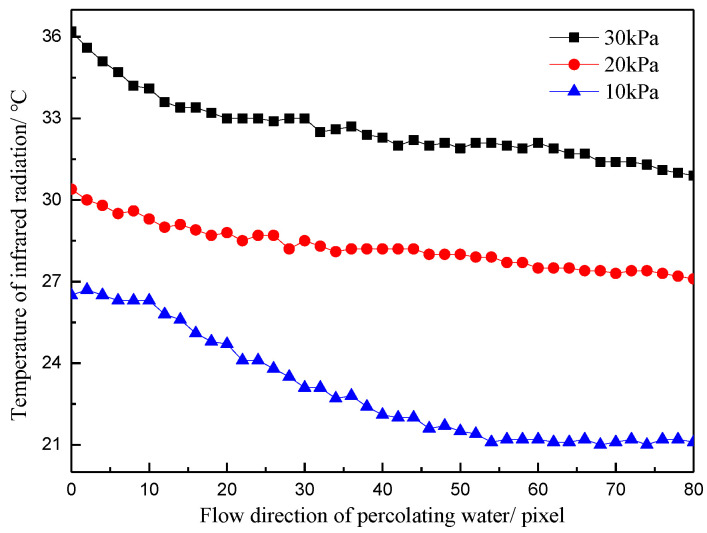
Temperature distribution curve of different water pressures along the flow direction.

The temperature division laws along the cross-sectional direction of the seepage water flow and along the water flow direction at the leakage point under the same water temperature and different water pressures are shown in Figure 5 and Figure 6.

As presented in Figure 5, when the water pressure was 10 kPa, the maximum surface temperature difference along the water flow cross-section direction was 27.1 °C, the minimum temperature difference was 19.3 °C, and the average temperature difference was 24.2 °C. When the water pressure was 30 kPa, the maximum surface temperature difference along the water flow cross-section direction was 27.5 °C, the minimum temperature difference was 9.0 °C, and the average temperature difference was 19.5 °C. The temperature field distribution along the cross-section of the seepage water at different seepage water pressures was symmetrical. The temperature of the water flowing through the concrete surface was inversely proportional to the distance to the water outlet point. The smaller the distance from the water outlet point was, the higher the temperature was. The temperature difference near the water outlet point increased continuously with the increase in leakage water pressure, which was proportional to it. The greater the seepage pressure was, the more drastic the temperature field changes were from the outlet point to the edge of the concrete specimen, and the greater the temperature difference was. The temperature changes tended to be flat at the edges.

Figure 6 shows the temperature distribution curve along the flow direction under the same water temperature and different water pressures. When the water pressure was 10 kPa, the maximum temperature difference along the water flow direction was 25.7 °C, the minimum temperature difference was 17.9 °C, and the average temperature difference was 23.4 °C. When the water pressure was 30 kPa, the maximum temperature difference along the water flow direction was 30.1 °C, the minimum temperature difference was 17.0 °C, and the average temperature difference was 29.7 °C. The concrete surface temperature was inversely proportional to the distance to the water outlet point. Similarly, the smaller the distance from the water outlet point was, the higher the temperature was and the greater the temperature change was.

(2) Influence of seepage water temperature on the temperature field

**Figure 7:**
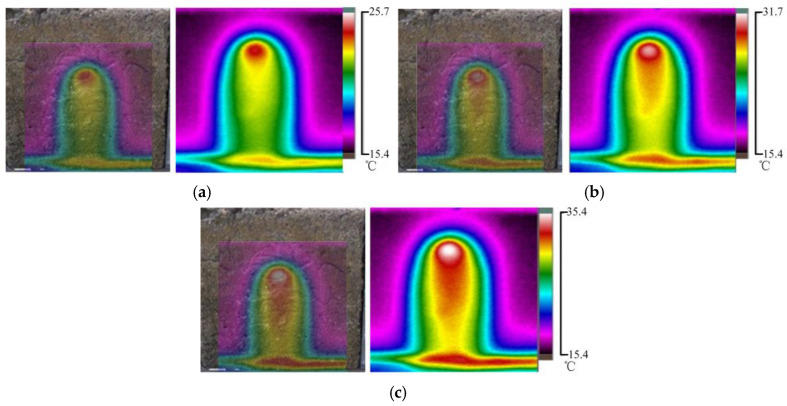
Temperature field of concrete surface under different seepage temperatures; (**a**) The leakage water temperature is 35 °C; (**b**) The leakage water temperature is 40 °C; (**c**) The leakage water temperature is 45 °C.

To study the influence of seepage water temperature on the temperature field distribution of the concrete specimen, three experimental conditions were set up to simulate the law of seepage water temperature change and concrete surface temperature field change. The water pressure in the three cases was 30 kPa, and the temperature of the leakage water was 35 °C, 40 °C, and 45 °C. The temperature field distribution of concrete surface under different seepage temperature fields is shown in Figure 7.

When the temperature of the leaking water was 35 °C, the highest temperature in the leaky area was 25.7 °C, and the temperature distribution of this part in the infrared thermal image was circular. With the increase in the distance to the leakage point, the temperature of the concrete sample was reduced, and the temperature distribution of this part showed a layered and elliptical distribution. When the temperature of the seepage water was 45 °C, the concrete temperature at the leakage point reached 35.4 °C. The high-temperature zone was distributed in a circular shape, and a high-temperature channel appeared at the bottom of the concrete sample. In the other regions, the distribution of the temperature field was similar to that at 35 °C.

**Figure 8:**
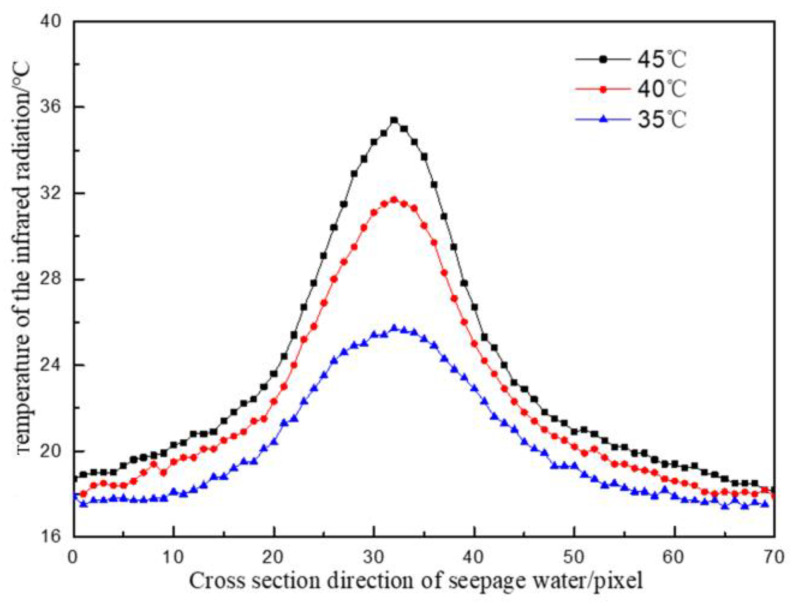
Temperature distribution curve along the cross-section direction of water flow at different temperatures.

**Figure 9:**
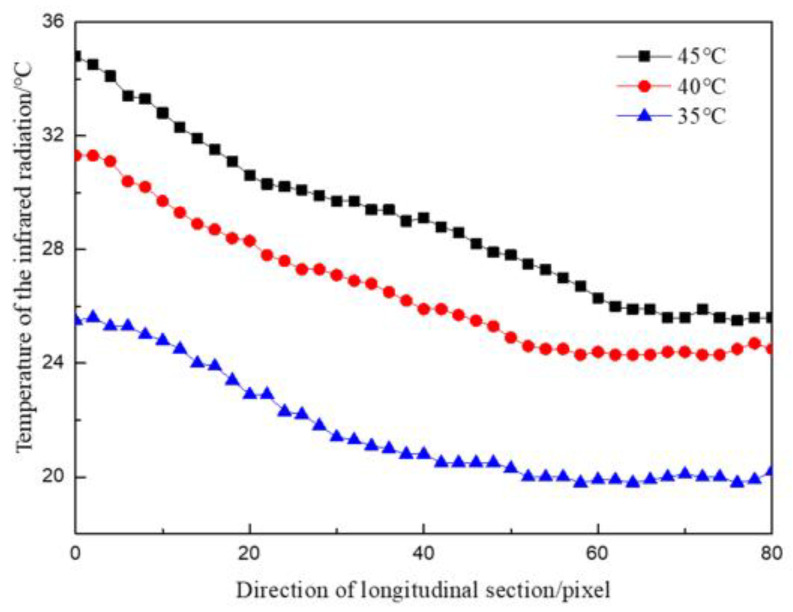
Temperature distribution curves along the flow direction at different temperatures.

Under the same water pressure and different leaking water temperatures, the temperature division law at the seepage point (the highest temperature) along the cross-section direction of the seepage flow and the direction of water flow is shown in Figure 8 and Figure 9.

As indicated in Figure 8, when the water temperature was 35 °C, the maximum temperature difference along the cross-section was 17.5 °C, the minimum temperature difference was 9.4 °C, and the average temperature difference was 14.8 °C. When the water temperature was 45 °C, the maximum temperature difference along the cross-section was 26.8 °C, the minimum temperature difference was 9.6 °C, and the average temperature difference was 21.8 °C. The temperature field distribution in the cross-section direction of the seepage water showed symmetry, similar to the result of the test on the influence of seepage water pressure on surface temperature; however, the temperature change near the outlet point was more intense than that in the test of the influence of seepage water pressure. From the water outlet to both ends of the concrete specimen, the temperature change gradually became flat. The higher the leaking water temperature was, the more drastic the temperature field changes were from the outlet point to both ends of the concrete block, and the greater the temperature difference was.

As shown in Figure 9, when the water temperature was 35 °C, the maximum surface temperature difference along the flow direction was 15.2 °C, the minimum temperature difference was 9.5 °C, and the average temperature difference was 13.4 °C. When the water temperature was 45 °C, the maximum surface temperature difference along the flow direction was 19.4 °C, the minimum temperature difference was 10.2 °C, and the average temperature difference was 16.0 °C. The concrete surface temperature was inversely proportional to the distance to the water outlet point. The smaller the distance from the water outlet point was, the higher the temperature was. The infrared thermal image of each condition showed that the highest temperature value appeared at the water outlet point.

### 3.2. Analysis of the Simulation Test Results on Concrete-Penetrating Water Hazards

(1) Influence of water pressure on the surface temperature field of permeable water

**Figure 10:**
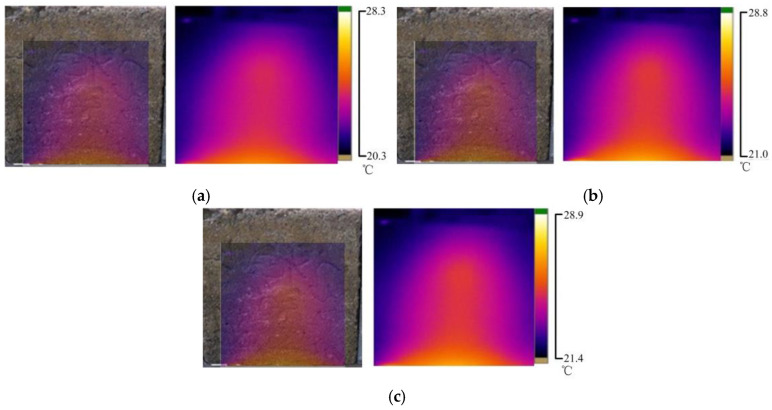
Temperature field distribution of concrete surface under different water pressures; (**a**) The water pressure is 10 kPa; (**b**) The water pressure is 20 kPa; (**c**) The water pressure is 30 kPa.

To study the influence of water temperature change at the penetration point on the surface temperature field distribution of the concrete specimen, three experimental conditions were established to simulate the influence of permeating water with different water pressures on the temperature field of the concrete surface. The temperature of the permeating water was 45 °C under the three conditions, and the pressure of the permeating water was 10, 20, and 30 kPa. The distribution of the concrete surface temperature field under different water pressures is shown in Figure 10.

As indicated in Figure 10, the temperature field distribution rules of the concrete surface were similar under the same and different temperatures of the permeating water. The temperatures were low in the upper-left and upper-right corners of the concrete sample. When the permeating water pressure was 10 kPa, the lowest temperature distribution was in the range of 20.3–21.9 °C. When the permeating water pressure was 30 kPa, the lowest temperature distribution was in the range of 21.4–22.8 °C. As indicated in Figure 10, the area with a high temperature showed a parabolic distribution, and the higher the initial permeating water pressure was, the larger the area of the high-temperature region was. In the defective part of the back, the temperature of the concrete sample was the highest, but the highest temperature of the concrete sample was about 28 °C under different water pressures. These phenomena indicate that the temperature difference of permeating water was related only to the effective permeable path. However, the permeable range was affected by the pressure of permeating water; the greater the pressure of permeating water was, the greater the permeable range was.

**Figure 11:**
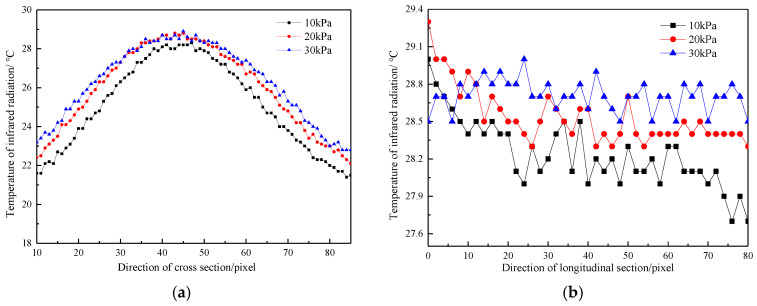
Temperature distribution curves along transverse and longitudinal sections under different water pressures; (**a**) Temperature distribution curve along transverse section; (**b**) Temperature distribution curve along the longitudinal section.

Under the condition of the same temperature and different water pressures, the temperature distribution laws along the cross-section direction of permeable water flow and along the water flow direction are shown in Figure 11a,b.

As indicated in Figure 11a, when the initial water pressure was 10 kPa, the maximum surface temperature difference along the cross-section was 24.7 °C, the minimum temperature difference was 16.7 °C, and the average temperature difference was 21.0 °C. When the initial water pressure was 30 kPa, the maximum surface temperature difference along the cross-section was 23.6 °C, the minimum temperature difference was 16.1 °C, and the average temperature difference was 19.5 °C. At this time, the distribution of infrared radiation temperature along the cross-section was symmetrical, and the curves were similar in shape (all of them were parabolic). The maximum temperature appeared at the hidden defect parts of the concrete specimen, and the temperature gradually decreased from the hidden defect surface to both ends of the concrete specimen.

When the initial water pressure was 10 kPa, the maximum surface temperature difference along the longitudinal section of the flow was 17.4 °C, the minimum temperature difference was 16.0 °C, and the average temperature difference was 16.8 °C. When the initial water pressure was 30 kPa, the maximum surface temperature difference along the longitudinal section of the flow was 16.5 °C, the minimum temperature difference was 16.0 °C, and the average temperature difference was 16.3 °C. In the longitudinal section direction, the infrared radiation temperature of the concrete sample was distributed stably and fluctuated, and the maximum temperature appeared in the hidden parts of the quality defects of the concrete specimen. The greater the water pressure was, the more drastic the temperature field change of the concrete sample was and the greater the temperature difference was. The closer the location was to the outlet point, the larger the temperature difference of the unit pixel points was.

(2) Influence of temperature on the temperature field in the permeable region

**Figure 12:**
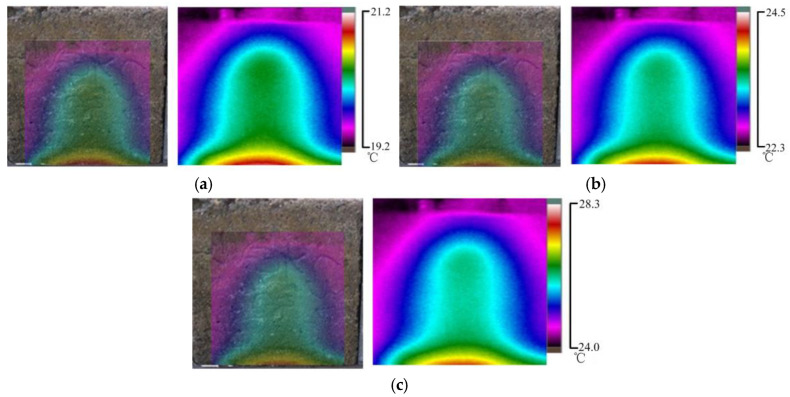
Temperature field distribution of concrete surface at different water temperatures; (**a**) The leakage water temperature is 35 °C; (**b**) The leakage water temperature is 40 °C; (**c**) The leakage water temperature is 45 °C.

This section studied the surface temperature distribution of concrete samples under different water temperatures with an initial water pressure of 30 kPa. The water temperature in Figure 12a is 35 °C, that in Figure 12b is 40 °C, and that in Figure 12c is 45 °C.

According to Figure 12a–c, under the same water pressure, the higher the permeable water temperature was, the higher the infrared radiation temperature of the concrete sample surface was. The surface temperature distributions of the concrete samples were similar under different penetration water temperatures. The area with a high temperature showed a parabolic and layered distribution, and the temperature gradually increased from top to bottom. The core region at the bottom of the sample had the highest temperature, and with the increase in the temperature of permeable water, the temperature of the core region also increased considerably, but the area of the high-temperature region decreased. The abovementioned phenomenon further confirms that the infrared radiation temperature on the surface of the concrete sample was related only to the temperature of the permeating water and the permeation path and was independent of pressure.

**Figure 13:**
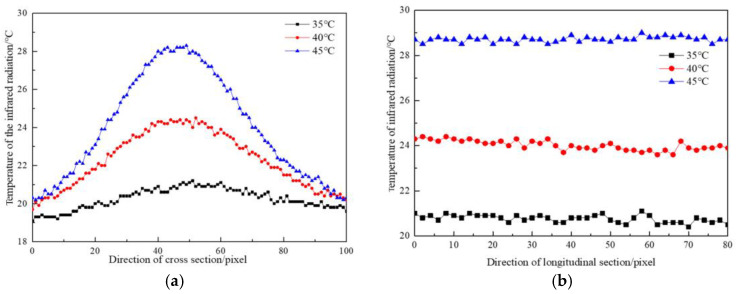
Temperature distribution curves along transverse and longitudinal sections at different temperatures; (**a**) Temperature distribution curve along transverse section; (**b**) Temperature distribution curve along the longitudinal section.

The temperature division laws along the cross-section direction of the permeable water flow and along the water flow direction under the same water pressure and different temperatures are shown in Figure 13a,b.

As indicated in Figure 13a, when the permeable water temperature was 35 °C, the maximum surface temperature difference along the cross-section of the flow was 15.4 °C, the minimum temperature difference was 13.9 °C, and the average temperature difference was 14.8 °C. When the permeable water temperature was 45 °C, the maximum surface temperature difference along the cross-section of the flow was 24.8 °C, the minimum temperature difference was 16.7 °C, and the average temperature difference was 21.0 °C. The maximum and average temperature differences were lower than those in the water leakage test. Figure 13a indicates that in the cross-section direction, the temperature decreased from the defective part of the concrete specimen to both sides, and the curve was distributed in a parabolic shape.

According to Figure 13b, when the permeable water temperature was 35 °C, the maximum surface temperature difference along the longitudinal section was 14.7 °C, the minimum temperature difference was 14.0 °C, and the average temperature difference was 14.3 °C. When the permeable water temperature was 45 °C, the maximum surface temperature difference along the longitudinal section was 21.2 °C, the minimum temperature difference was 20.7 °C, and the average temperature difference was 21.0 °C. The temperature change trend in the vertical section direction was relatively stable. When the water temperature was 35 °C, the stable fluctuation of infrared radiation was 0.7 °C. When the water temperature was 40 °C, the stable fluctuation of infrared radiation was only 0.8 °C.

### 3.3. Discussions

The research results of the laboratory experiments carried out in this paper were similar to Bhalla [28], and the temperature difference obtained by the field thermal imaging shot in this paper is similar to Antoine [29]. As a non-contact sensing method, ITNDT has many advantages, such as intuitive image, fast operation, and strong mobility. However, thermal imaging technology still had certain defects. For example, thermal imaging was mainly applied to the large temperature difference between the detected target and the background, and compared with distributed optical fiber leak detection technology, thermal imaging temperature measurement technology had poor coverage. Combining distributed optical fiber temperature measurement technology with infrared thermal imaging technology was the research content of the next stage of this paper.

## 4. Applications

### 4.1. Deep Foundation Pit Project at Shanghai Expo Area

#### 4.1.1. Project Profile

**Figure 14:**
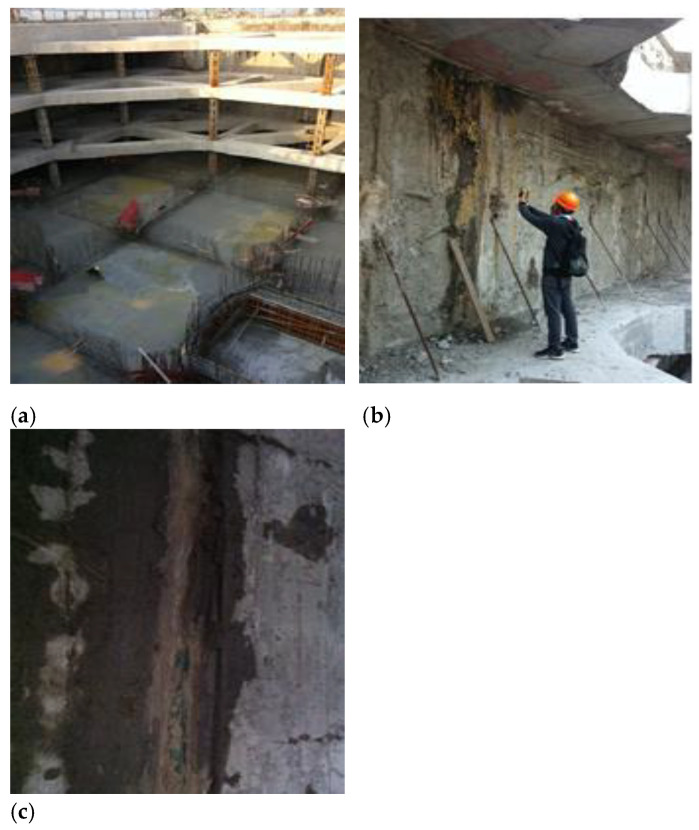
Photos of foundation pit site and water leakage detection; (**a**) Foundation pit overview; (**b**) The joint of the diaphragm wall; (**c**) Seepage of diaphragm wall.

A deep foundation pit project at Shanghai World Expo was examined in this study. As shown in Figure 14, the foundation pit project has three underground floors. The excavation depth of the foundation pit is 16 m, the length from east to west is about 260 m, the length from north to south is about 160 m, and the excavation area of the foundation pit is 30,000 m^2^. The surrounding environment of the foundation pit is relatively complicated. The nearest distance between the southeast corner gas pipe and the foundation pit boundary line is about 3.5 m. The south side is adjacent to the road, which is buried under the gas, rainwater, and sewage pipelines. The nearest pit line is about 6 m. The west side is adjacent to the subway tunnel, so the excavation of the foundation pit requires extremely high deformation.

The foundation pit adopts the slab support system (diaphragm wall), and three horizontal concrete supports are set horizontally. Groundwater seeps into the diaphragm wall from the gap because the mud was not removed during the construction at the joints of the diaphragm wall. Sometimes, point, line, and surface leakages can be identified by the naked eye. They can be divided into water seepage, water leakage, and mud water in accordance with the different water outputs. Sometimes, the water in the quality defect site does not penetrate into the surface of the diaphragm wall; that is, the groundwater penetrates into the diaphragm wall but not into its inner surface. In this case, a potential leakage risk exists.

#### 4.1.2. Analysis of the Infrared Radiation Temperature of the Leakage at the Joint of the Diaphragm Wall

**Figure 15:**
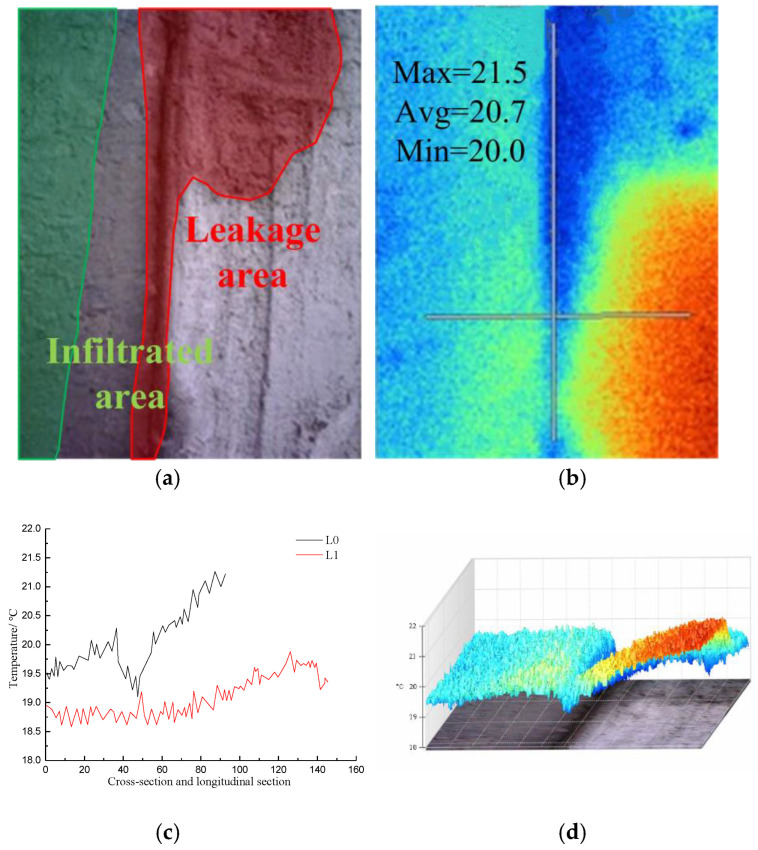
Infrared image and data analysis of leakage at the joint of diaphragm wall; (**a**) Visible light photograph; (**b**) Infrared radiation image; (**c**) Temperature distribution curves of cross-section and longitudinal section at the joint of diaphragm wall; (**d**) 3D-IR image.

Figure 15 shows visible light photos, infrared radiation images, temperature distribution curves in the cross-section and longitudinal section of the diaphragm wall joints, and 3D-IR images of the leakage area of the diaphragm wall joints.

The infrared image of the joint indicates that the temperature range within the image was 18.4–21.6 °C, and the average temperature was 19.8 °C. The temperature of the leakage area was the lowest, namely, 18.6 °C. Along the direction of joint cross-section L0, the maximum temperature was 21.3 °C, the minimum temperature was 19.1 °C, and the average temperature was 20.1 °C. The lowest temperature appeared at the joint. The temperature decreased gradually from the seam to the left end and increased gradually to the right end. Along the L1 direction of the joint longitudinal section, the maximum temperature was 19.9 °C, the minimum temperature was 18.6 °C, and the average temperature was 19.1 °C. The temperature tended to increase along the longitudinal section L1 of the joint. The 3D-IR image showed that the temperature at the seam soaked by water was the lowest. The wall on the left side of the joint was soaked by water, and some areas on the right wall were saturated with water. The average temperature on the left wall was lower than that on the right wall.

#### 4.1.3. Infrared Radiation Temperature Analysis of Potential Water Leakage at the Joint of the Diaphragm Wall

**Figure 16:**
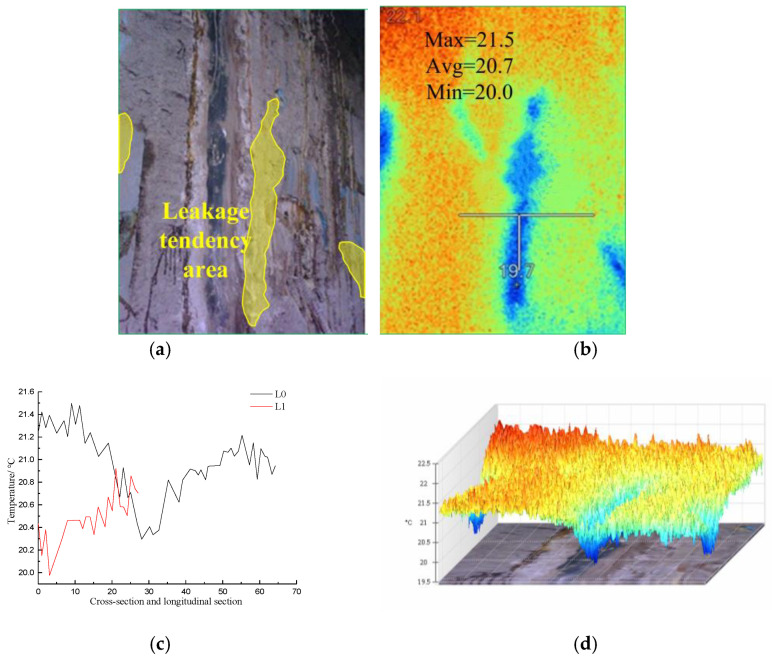
Infrared image temperature data analysis of hidden danger of penetration areas in diaphragm wall joint; (**a**) Visible light photograph; (**b**) Infrared radiation image; (**c**) Temperature distribution curves of cross-section and longitudinal section at the joint of diaphragm wall; (**d**) 3D-IR image.

Figure 16 shows visible light photos, infrared radiation images, temperature distribution curves in the cross-section and longitudinal section of the diaphragm wall joints, and 3D-IR images of the leakage area with the hidden danger of penetration areas on the diaphragm wall joints.

The visible light images of the joint of the diaphragm wall in Figure 15 show a leakage. The water outlet was sealed with soluble polyurethane after reinforcement, so the leakage was controlled. The infrared radiation images indicated that the temperature range within the image was 19.7–22.1 °C, and the average temperature was 21.1 °C. Along the direction of joint cross-section L0, the maximum temperature was 21.5 °C, the minimum temperature was 20.3 °C, and the average temperature was 21 °C. The lowest temperature appeared at the joint, and the lowest temperature was 19.7 °C. The heat conduction coefficient of the part sealed by the soluble polyurethane was different from that of the intact part of the wall, which resulted in an abnormal temperature distribution on the surface of the diaphragm wall. Along the direction of the joint longitudinal section L1, the maximum temperature was 20.9 °C, the minimum temperature was 20 °C, and the average temperature was 20.5 °C. The temperature increased along the direction of the longitudinal section L1 of the joint. The 3D-IR image indicated that the surface temperature of the area blocked by soluble polyurethane was the lowest.

### 4.2. Deep Foundation Pit of Hanzhong Road Station of Shanghai Subway Line 13

#### 4.2.1. Project Profile

**Figure 17:**
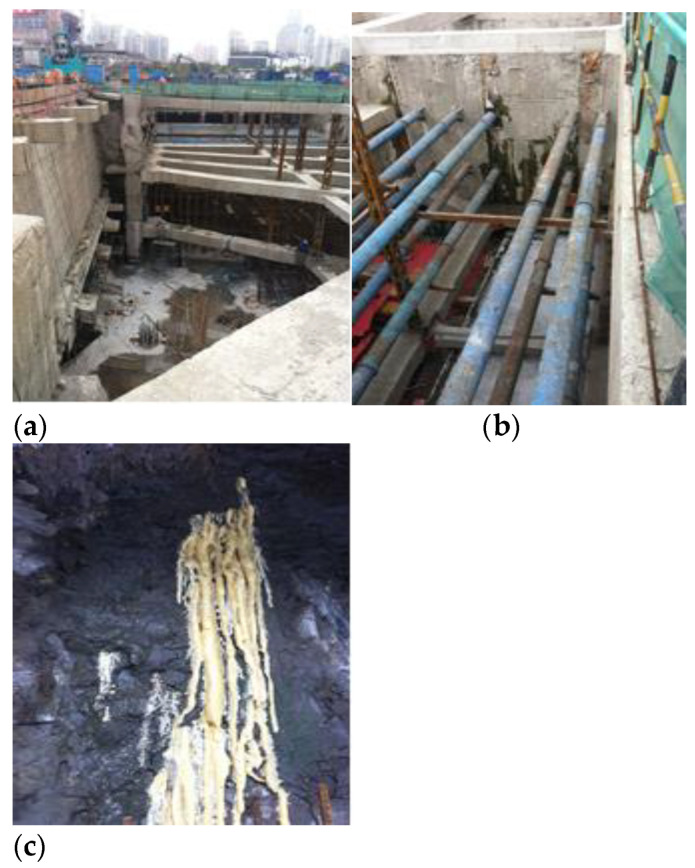
Photos of foundation pit site and water leakage detection; (**a**) Foundation pit overview; (**b**) Supporting structure of foundation pit; (**c**) The seepage of the foundation pit.

Hanzhong Road Station of Shanghai Subway Line 13 is composed of two foundation pits of Lines 12 and 13, one foundation pit for the equipment room, and one foundation pit for the transfer hall, as shown in Figure 17. The total construction area is 53,693 m^2^. The project is the deepest rail transit hub station in Shanghai at present. The station of Line 13 is an island station with five underground floors, and the excavation depth is 31–33 m. The enclosure structure is a diaphragm wall, steel support, and concrete support. A total of nine supports are present, as shown in Figure 16. The second, third, fifth, seventh, and ninth are steel supports, and the first, fourth, and sixth are concrete supports.

#### 4.2.2. Analysis of Infrared Radiation Temperature for the Leakage of the Diaphragm Wall

**Figure 18:**
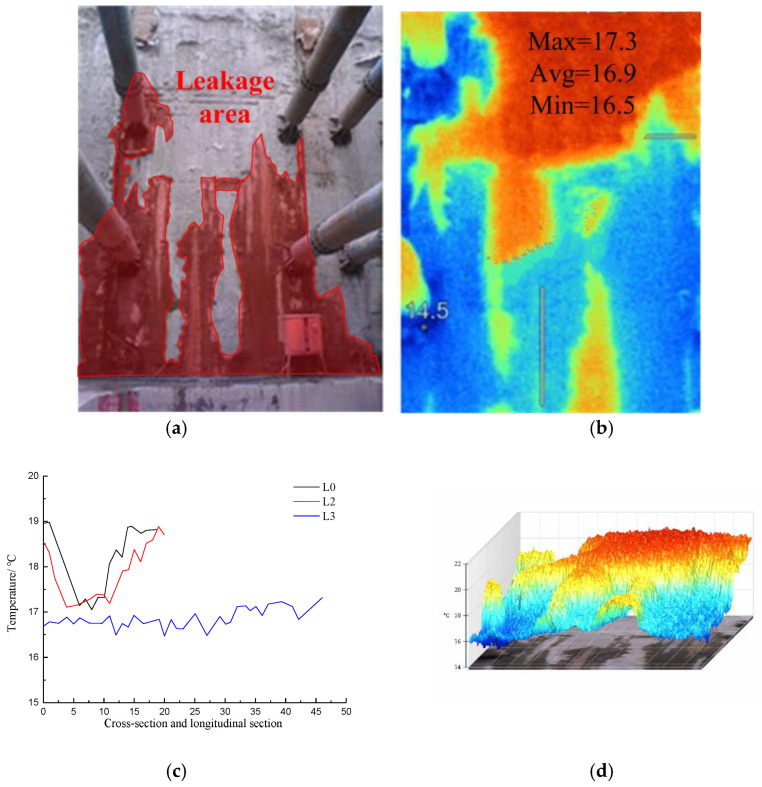
Analysis of infrared image temperature data of leakage of diaphragm wall; (**a**) Visible light photograph; (**b**) Infrared radiation image; (**c**) Temperature distribution curves of cross-section and longitudinal section at the diaphragm wall; (**d**) 3D-IR image.

Figure 18 shows visible light photos, infrared radiation images, temperature distribution curves in the cross-section and longitudinal section, and 3D-IR images of the leakage area of the diaphragm wall.

The infrared image of the diaphragm wall indicates that the temperatures within the image range were 14.5–21.1 °C, and the average temperature was 17.8 °C. The high-temperature and low-temperature areas crossed each other. Along the L0 direction, the maximum temperature was 19 °C, the minimum temperature was 17.1 °C, and the average temperature was 18.2 °C. The lowest temperature of 17.1 °C appeared in the leakage area. Along the direction of L2, the maximum temperature was 18.9 °C, the minimum temperature was 17.1 °C, and the average temperature was 17.8 °C. The lowest temperature of 17.1 °C occurred in the leakage area. Along the direction of longitudinal section L3 of the seepage area, the maximum temperature was 17.3 °C, the minimum temperature was 16.5 °C, and the average temperature was 16.9 °C. The temperature tended to increase along the direction of longitudinal section L3 of the seepage area because the surface temperature of concrete was higher than the leakage temperature, and the water flow continuously absorbed heat in the flowing process. The 3D-IR images indicate that the temperature of the image area affected by point leakage was lower than that of the normal area, and the area affected by point leakage gradually expanded from top to bottom.

#### 4.2.3. Analysis of the Infrared Radiation Temperature in the Penetration Area of the Diaphragm Wall

**Figure 19:**
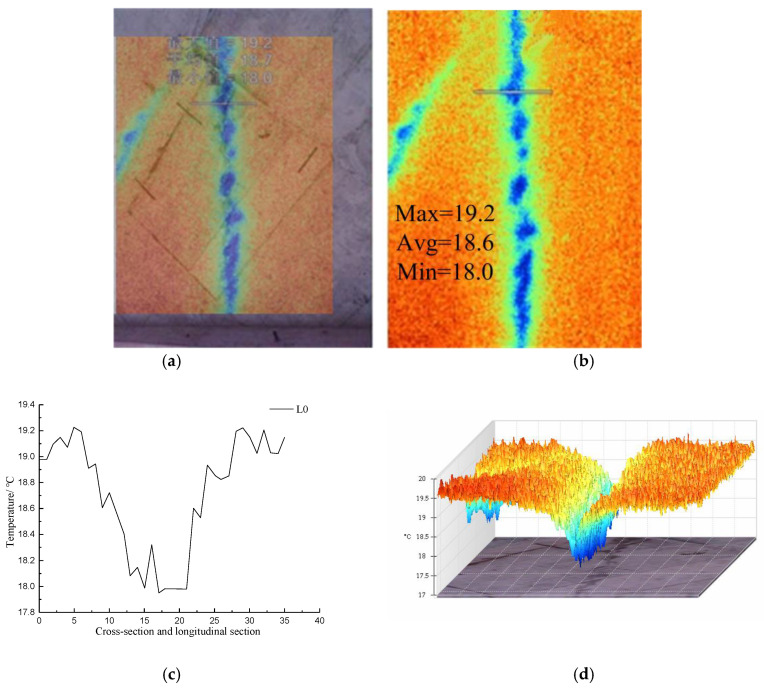
Analysis of infrared image temperature data in latent area of foundation pit seepage water; (**a**) Visible light photograph; (**b**) Infrared radiation image; (**c**) Temperature distribution curves of cross-section and longitudinal section at the joint of diaphragm wall; (**d**) 3D-IR image.

Figure 19 shows visible light photos, infrared radiation images, temperature distribution curves in the cross-section and longitudinal section of the diaphragm wall joints, and 3D-IR images of the leakage area of the hidden danger of penetration areas on the diaphragm wall.

As shown in Figure 19a, a line leakage had occurred in the enclosing purlin of the upper layer. A week later, during the second shoot, the leakage had stopped, and the water marks left by the leakage were still visible. The infrared radiation image shows that the temperature within the image range was 17.5–19.9 °C, the average temperature was 19.1 °C, the temperature in the penetration area was the lowest (i.e., 18.0 °C), and the distribution was linear. Along the direction of cross-section L0, the maximum temperature was 19.2 °C, the minimum temperature was 18 °C, the average temperature was 18.7 °C, and the lowest temperature (i.e., 18 °C) appeared in the permeable water. The heat conduction coefficient of the linear area saturated with groundwater was different from that of the intact part of the diaphragm wall, which resulted in an abnormal distribution of the surface temperature of the diaphragm wall. The temperature curve along the direction of L0 was symmetrical. The 3D-IR image shows that the surface temperature of the area where the leakage occurred was the lowest.

## 5. Conclusions

To overcome the limitation of manual detection of diaphragm wall leakage, thermal imaging technology was used to detect the leakage area and potential leakage area of diaphragm walls. The distribution of the temperature field on the concrete surface under different temperatures and pressures of water was studied using laboratory experiments. The thermal imaging technology was used in deep foundation pits. The following conclusions were reached:

(1) The infrared thermal imaging method can detect the seepage penetration tendency and potential leakage area, which cannot be identified by the naked eye intuitively, objectively, and accurately.
(2) In the leakage area, the higher the water pressure and temperature, the higher the temperature of the penetration point area. The temperature difference at the leakage point was about 10 °C under different permeable water temperatures.
(3) In the potential leakage area, the temperature at the seepage point was the same under different seepage water pressures; the higher the temperature of the seepage water, the greater the temperature difference at the seepage point.
(4) Although no trace of water was observed on the concrete surface, hidden quality problems inside the concrete led to groundwater infiltration, resulting in the surface temperature of the hidden defect being 1.2~1.5 °C lower than the surrounding wall.
(5) According to the above research, infrared thermal imaging technology can accurately determine the leakage area and leakage tendency area of the underground diaphragm wall.

## Figures and Tables

**Table 1:** Test conditions.

Simulate the Type of Water Seepage	Control Condition 1	Condition 2
Permeation test	The water temperature is kept at 45 °C	Water pressure is 10 kPa
		Water pressure is 20 kPa
		Water pressure is 30 kPa
	The water pressure is maintained at 30 kPa	Water temperature is 35 °C
		Water temperature is 40 °C
		Water temperature is 45 °C
Permeation tendency area	The water temperature is kept at 45 °C	Water pressure is 10 kPa
		Water pressure is 20 kPa
		Water pressure is 30 kPa
	The water pressure is maintained at 30 kPa	Water temperature is 35 °C
		Water temperature is 40 °C
		Water temperature is 45 °C

## Data Availability

Data presented in this study are available on request from the corresponding author.
